# Genetic and Cytological Analyses of the Natural Variation of Seed Number per Pod in Rapeseed (*Brassica napus* L.)

**DOI:** 10.3389/fpls.2017.01890

**Published:** 2017-11-02

**Authors:** Yuhua Yang, Ying Wang, Jiepeng Zhan, Jiaqin Shi, Xinfa Wang, Guihua Liu, Hanzhong Wang

**Affiliations:** Key Laboratory of Biology and Genetic Improvement of Oil Crops, Ministry of Agriculture, Oil Crops Research Institute of the Chinese Academy of Agricultural Sciences, Wuhan, China

**Keywords:** *Brassica napus*, seed number per pod, maternal effect, embryo effect, cytoplasm effect, ovule number, ovule fertility, fertilization

## Abstract

Seed number is one of the key traits related to plant evolution/domestication and crop improvement/breeding. In rapeseed germplasm, the seed number per pod (SNPP) shows a very wide variation from several to nearly 30; however, the underlying causations/mechanisms for this variation are poorly known. In the current study, the genetic and cytological bases for the natural variation of SNPP in rapeseed was firstly and systematically investigated using the representative four high-SNPP and five low-SNPP lines. The results of self- or cross-pollination experiment between the high- and low-SNPP lines showed that the natural variation of SNPP was mainly controlled by maternal effect (mean = 0.79), followed by paternal effect (mean = 0.21). Analysis of the data using diploid seed embryo–cytoplasmic–maternal model further showed that the maternal genotype, embryo, and cytoplasm effects, respectively, explained 47.6, 35.2, and 7.5% of the genetic variance. In addition, the analysis of combining ability showed that for the SNPP of hybrid F_1_ was mainly determined by the general combining ability of parents (63.0%), followed by special combining ability of parental combination (37.0%). More importantly, the cytological observation showed that the SNPP difference between the high- and low-SNPP lines was attributable to the accumulative differences in its components. Of which, the number of ovules, the proportion of fertile ovules, the proportion of fertile ovules to be fertilized, and the proportion of fertilized ovules to develop into seeds accounted for 30.7, 18.2, 7.1, and 43.9%, respectively. The accordant results of both genetic and cytological analyses provide solid evidences and systematic insights to further understand the mechanisms underlying the natural variation of SNPP, which will facilitate the development of high-yield cultivars in rapeseed.

## Introduction

Seed number is one of the key traits related to plant evolution and domestication, because species/accessions/individuals with more offsprings/seeds are more advantageous in reproduction, and also preferentially to be selected and maintained by ancient human (Gnan et al., 2014). From the applied perspective, seed number is one of the most important factors of yield composition and also one of the major targets for genetic improvement in most crops, including rapeseed (Yang et al., 2016).

As for rapeseed, seed number per pod (SNPP), together with pod number and seed weight are the three components of yield. Although the three yield components of rapeseed have different degree of negative correlation, the correlation coefficients are usually small (Shi et al., 2009), which means that the final yield can be improved by increasing the individual yield components (such as SNPP). As expected, SNPP usually showed the relatively high positive correlation with seed yield, although the coefficients varied in the different studies (Zhang et al., 2012). The numbers of seeds per pod (≈20) for the current rapeseed cultivars are much lower than its maximum (≈30) in the germplasm resources (Chen, 2014), which suggests a great potential of the genetic improvement of SNPP in rapeseed. The SNPP in rapeseed is multiply determined by its components *t*, including the number of ovules per ovary, the proportion of ovules to be fertilized (=the proportion of fertile ovules × the proportion of fertile ovules to be fertilized), and the proportion of fertilized ovules to develop into seeds. In rapeseed, the number of ovules per ovary is determined by the ovule differentiation process (Yang et al., 2016). The proportion of ovules to be fertilized is determined at the fertilization process, which is dependent on many factors such as ovule fertility, pollen sterility, the interaction between the pollen grain/pollen tube including the sperm cells with the various sporophytic maternal tissues, and the cells of the female gametophyte (Dresselhaus and Franklin-Tong, 2013; Li et al., 2014; Li S.P. et al., 2015). The proportion of fertilized ovules to develop into seeds is determined by the seed development process, which is affected by both internal (the nutritional and physiological status of the mother plant) and external (environmental conditions) factors as well as their interaction (Li et al., 2014; Xu et al., 2014). The main biological processes that determine SNPP are basically clear, however, which processes might be responsible for its natural variation in germplasm resource are poorly known.

For most of the major crops in the world^1^, seed is the main harvested/utilized organ for human. As mostly heterotrophic organ, seed consists of three components: a diploid embryo, triploid endosperm, and diploid seed coat (Li and Li, 2015). In addition, the development of a seed is dependent on the nutrients supplied by the mother plant (Hua et al., 2012). Therefore, the genetic model of the seed traits in theory includes the effects of the maternal genotype, cytoplasm, embryo, and endosperm (Zhu, 1997), which also can be attributed to maternal and xenia effects (Li N. et al., 2015). Despite their fundamental importance in genetic and breeding studies, the relative contributions of these effects to SNPP are poorly known or completely unclear in rapeseed, because the previous genetic studies of SNPP were focus on the additive–dominant–epistatic model (Li and Gu, 1992; Wei, 2000; Zhang et al., 2008, 2010).

In the present study, nine highly pure rapeseed lines with large SNPP variation and broad genetic background were used to systematically dissect the genetic and cytological causes of the natural variation of SNPP in rapeseed. In detail, the main objectives were to (1) select several representative lines for further study on SNPP, (2) estimate the maternal and xenia effects on SNPP, (3) estimate the genetic effects of maternal genotype and those of cytoplasm, embryo, and endosperm on SNPP, (4) estimate the combining ability of SNPP, and (5) investigate the cytological causes of the natural variation of SNPP in rapeseed.

## Materials and Methods

### Plant Materials

A collection of 1063 rapeseed lines/cultivars from all over the world was constructed and preserved in our lab (Li N. et al., 2015). This collection was genotyped using 118 published SSR (simple sequence repeat) markers (Shi et al., 2014) that are evenly distributed across all the 19 rapeseed chromosomes and investigated for tens of important traits. Of these, four high-SNPP (No. 02395, SWU63, WH85, and Zhongshuang11) and five low-SNPP (Campina, No. 4531, No. 73290, No. 93237, and No. 96016) lines with broad genetic background were selected for further study.

### Genetic Diversity Analysis

The gene diversity, heterozygosity, polymorphic information content (PIC), and genetic distance among the lines were calculated using Powermarker V3.25 software (Liu and Muse, 2005). A dendrogram was constructed based on the UPGMA (unweighted pair group method with arithmetic mean) algorithm implemented in NTSYSpc 2.10e software (Rolf, 2000). The relative kinship coefficients were calculated using the SPAGeDi software package (Hardy and Vekemans, 2002). All the negative values between these lines were set to 0 (Yu et al., 2006).

### Maternal vs. Xenia Effects Study

For the maternal effect study, we performed the emasculation and self- or cross-pollination between the high- and low-SNPP lines with minimal environmental influences as described in our previous study (Wang et al., 2010). The four high-SNPP (No. 02395, SWU63, WH85, and Zhongshuang11) and five low-SNPP (Campina, No. 4531, No. 73290, No. 93237, and No. 96016) lines were used for this study in March 2014 in Wuhan (code W14). In March 2015, the cross-pollination of the high-SNPP line No. 02395 with other five low-SNPP lines was repeated in Wuhan (code W15). All of the detailed procedures were performed as described in our previous study (Li N. et al., 2015). At maturity, the pods were harvested and threshed to measure the number of seeds per pod.

### Dynamic Observation of the Number of Ovules/Seeds per Ovary/Pod

The number of ovules per ovary of rapeseed was observed only at the date of flowering, because the previous studies showed that the number should be stable during the ovule development (Li et al., 2014). The dynamic observation was conducted in five stages, i.e., 0, 7, 15, and 25 days after flowering (DAF), and at maturity. To maintain the developmental identity of the samples for different lines, flowers on middle of the main inflorescence and with the same flowering date were marked with red ropes. At each stage, three flowers/pods were sampled from the marked positions of 10 representative plants and used to observe the number of ovules/seeds per ovary/pod. The detailed procedures were performed as our previous study (Yang et al., 2016).

### Observation of Cytological Characteristics Related to Ovule and Pollen Fertility as well as Fertilization

To investigate the fertility of ovule, the morphology and structure of embryo sac at flowering were observed by serial paraffin sections. For each line, three buds at 0-DAF were sampled from the main inflorescence of 10 representative individuals, respectively, and the pistils were quickly peeled off from the buds and fixed in 50% FAA solution. Then, the ovules were peeled off from the ovaries, dehydrated through an ethanol series of 75, 85, 95, and 100%, and embedded in the paraffin. The detailed experimental procedures for section were as described in the previous studies (Li et al., 2014; Yang et al., 2016). Generally, a total of 30–40 ovules have got the successful sections for each individual. For each ovule, a total of 30 serial sections were made and then observed using a microscope. To investigate the fertility of pollen, pollen viability (by acetic red dyeing) and pollen germination rate *in vitro* were observed. For each line, three flowers at 0-DAF were sampled from the main inflorescence of 10 representative individuals, respectively. To investigate the pollen viability, the pollens were quickly collected from the three sampled flowers of each individual, stained with 1% acetocarmine and then checked under a fluorescence microscope, according to their staining and shape. To investigate the pollen germination rate *in vitro*, the pollens were quickly shaken off from the three sampled flowers of each individual to spread evenly on the pollen germination medium, cultured at room temperature for 3 h, and then observed using the fluorescence microscope. The detailed experimental procedures were as described in the previous studies (Sato et al., 1998; Fan et al., 2012; Yang et al., 2016).

Pollen germination on the stigma and pollen tubes growth in the style and accession into ovary were observed. At 1- to 3-DAF, three pistils from the main inflorescence of 10 representative individuals were, respectively, sampled for each line and fixed in 50% FAA for 24 h, softened in 8 M NaOH at 65°C for 3 h, washed with 50 mM K-phosphate buffer (pH = 7.5), and stained in 0.1% aniline blue. The stained pistils and pollen tubes were observed using a fluorescence microscope. The detailed experimental procedures were as described in the previous studies (Chen and Chang, 1980; Xiao et al., 1995; Yang et al., 2016).

### Field Experiment and Trait Measurement

For the genetic and cytological analyses, the self-cross seeds of four high-SNPP and five low-SNPP lines were sown in 10 rows with 15 plants per row. To further estimate the cytoplasmic effect of number of seeds per pod, the F_1_ seeds of the reciprocal crosses were arranged in a randomized complete block design with three repetitions. Each block contained 1 row with 15 plants. The seeds were sown by hand, and the field management followed local standard agricultural practice.

For the four high-SNPP and five low-SNPP lines as well as the reciprocal hybrid F_1_ plants between them, number of seeds per pod was measured from the well-developed pods from the whole main raceme (natural pollination) of at least 15 representative individuals (Yang et al., 2016). For the maternal effect study, each of the well-developed pods obtained from the self- and cross-pollination were individually harvested and threshed to measure the number of seeds per pod.

### Statistical Analysis

Pearson’s correlation coefficient was calculated using the CORR procedure implemented in the SAS software (SAS Institute, 2000). The *t*-test function in the Microsoft Excel 2010 or ANOVA procedure in SAS V8 was, respectively, used to test the significance of statistical parameters between two or more lines/combinations. The maternal effect on SNPP was estimated using the method described in the previous studies (Cong, 1996; Wang et al., 2010). The calculated formula is as follows: *m* (the maternal effect value) = ∑ (F1 – P2)(P1 – P2)/∑ (P1 – P2)^2^, P_1_, P_2_, and F_1_ are the number of seeds per pod obtained from the self-pollination of female and male parents and cross-pollination between them, respectively.

The diploid seed embryo–cytoplasm–maternal (GoCGm) model (Zhu, 1997) was employed to determine the genetic main effects and their genotype by environment (*GE*) interaction effects using QGAStation 2.0 software package^2^. Genetic variance components were estimated according to the MINQUE (0/1) method (Zhu and Weir, 1994). The genetic main effects and *GE* interaction effects were predicted by the adjusted unbiased prediction (AUP) method (Zhu, 1996). The standard error of estimated variance was analyzed by the jackknife procedure.

The plot means were subjected to the combining ability analysis based on the following formulae (Li et al., 1997): X_ij_ = μ + G_i_ + G_j_ + S_ij_ + e, where μ is the overall mean value; *X*_ij_ is the phenotypic value of the F_1_ hybrid between the *i-*th and *j-*th parents; *G*_i_ and *G*_j_ are the general combining ability (GCA) of the *i-*th and *j*-th parents, respectively; *S*_ij_ is the special combining ability (SCA) between the *i*-th and *j*-th parents; e means the error.

## Results

### Phenotypic Variation of SNPP and Genetic Diversity Analysis for the Research Materials

According to the phenotypic and genotypic analyses (Shi et al., unpublished data) of a total of 1063 worldwide collected rapeseed lines preserved in our lab, nine ones were selected for further genetic and cytological analyses on SNPP.

**FIGURE 1:**
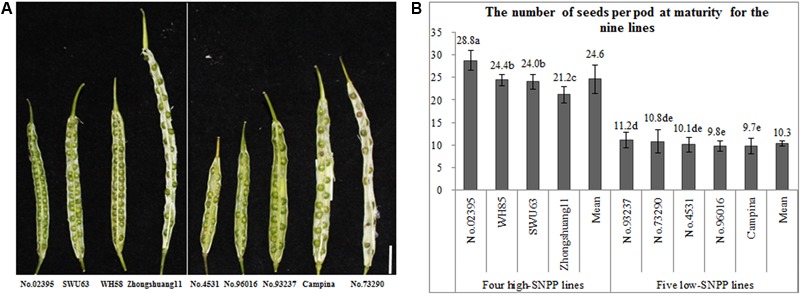
Variation of the number of seeds per pod among the nine rapeseed lines. **(A)** A representative pod from each of the nine lines, which was cut across the diaphragm, showing the difference in number of seeds per pod for these lines. Bar = 1 cm. **(B)** The multiple comparisons of the number of seeds per pod for the nine lines. The same lowercase letter indicates no significant difference at 0.05 probability level.

The numbers of seeds per pod of the nine rapeseed lines showed great (nearly threefold) variation ranging from 9.7 to 28.8, which can be divided into two types: four high-SNPP and five low-SNPP lines (**Figure 1**). The numbers of seeds per pod for the four high-SNPP (21.2–28.8) were all much higher than those (9.7–11.2) for the five low-SNPP lines, and the mean (24.6) of the former was also significantly (*P* = 0.0023) higher than that (10.3) of the latter.

**FIGURE 2:**
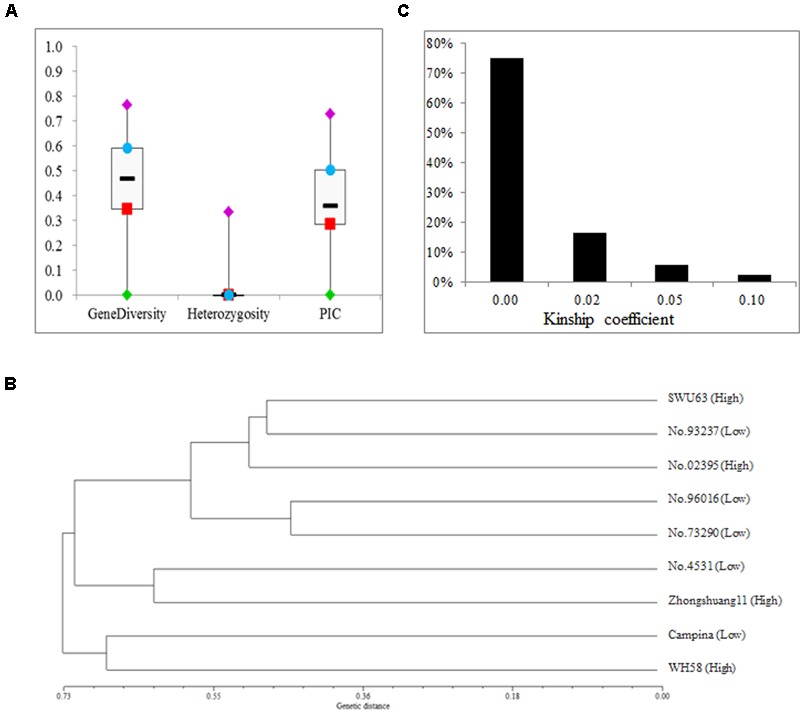
The genetic diversity, distance, and kindship of the nine rapeseed lines. **(A)** Summary statistics of genetic diversity and heterozygosity for the nine rapeseed lines. **(B)** A dendrogram of the nine rapeseed lines clustered on the basis of Nei’s genetic distance. **(C)** The distribution of kinship coefficients for the nine rapeseed lines.

The average heterozygosity of the nine lines was estimated using 118 SSR markers (Supplementary Table S1), which ranged from 0.00 to 0.04 with a mean of 0.02 (**Figure 2A**). For all these makers, the gene diversity and PIC varied from 0.01 to 0.77 and from 0.01 to 0.73, with an average of 0.43 and 0.37, respectively. The genetic distances among the nine lines ranged from 0.45 to 0.89, with an average of 0.66 (Supplementary Table S2). Then, a dendrogram was built with the UPGMA algorithm by the genetic distances among the nine lines (**Figure 2B**). As expected, there was no relationship between the classification and SNPP value. The kinship coefficients among the nine lines ranged from 0 and 0.13 with an average of 0.01 (**Figure 2C** and Supplementary Table S3). These results strongly suggested that the nine selected rapeseed lines should be highly pure and from broad genetic background.

### Maternal vs. Xenia Effects on SNPP

**FIGURE 3:**
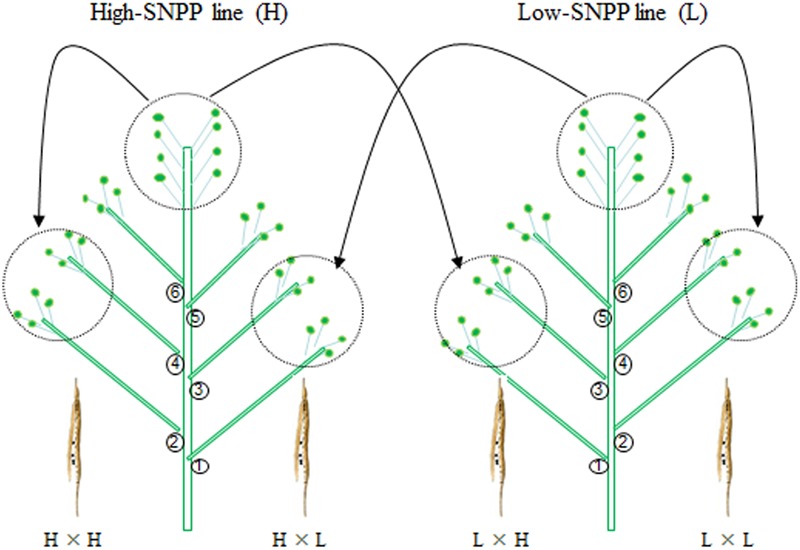
A schematic cartoon of the reciprocal crossing experiment used to estimate the maternal/xenia effect on seed number per pod (SNPP). For each pairwise combination between a high- and low-SNPP line, the alternant branches of two plants, respectively, from the high- and low-SNPP lines were emasculated and self- or cross-pollinated with each other at the same day to minimize the experimental error.

**FIGURE 4:**
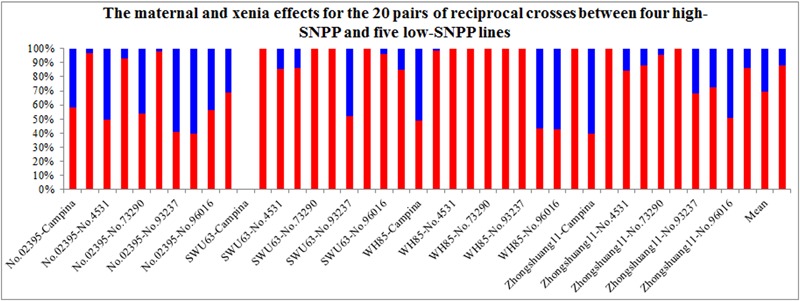
The maternal and xenia effects for the reciprocal crosses between the four high-SNPP and five low-SNPP lines. The horizontal axis shows the pairwise parental combinations between the four high-SNPP and five low-SNPP lines. The red and blue bars represent the maternal and xenia effects, respectively.

The maternal/xenia effect was estimated by emasculation and self- or cross-pollination experiment between the above mentioned four high-SNPP and five low-SNPP lines (**Figure 3**). For most (27; 69.2%) of the reciprocal crosses, the number of seeds per pod obtained from cross-pollination was similar to that of the female parent (self-pollination) (**Table 1**), and the calculated maternal effect was more than twice of the corresponding xenia effect (**Figure 4**). Whereas for the other 12 crosses (10 from H×L cross and 2 from L×H cross), the number of seeds per pod obtained from cross-pollination was near to the mean of the female and male parents (self-pollination), and the calculated maternal and xenia effects (0.40–0.60) were comparable. Overall, the average maternal and xenia effects of all the reciprocal crosses were 0.79 and 0.21, respectively. These results suggested that the SNPP difference between the high- and low-SNPP lines was mainly attributable to maternal effect, followed by xenia effect.

**Table 1:** The number of seeds per pod for the self- and cross-pollination between the four high-SNPP and five low-SNPP lines.

Line combinations (H–L)	H×H	H×L	L×L	L×H	Experiment code
No. 02395–No. 73290	24.2 ± 0.2c	16.6 ± 0.6b	7.7 ± 0.6a	8.1 ± 1.3a	W14, W15
No. 02395–No. 93237	24.8 ± 0.6d	16.8 ± 1.6b	11.3 ± 1.1a	19.4 ± 0.3c	W14, W15
No. 02395–Campina	23.4 ± 1.9c	17.3 ± 2.1b	8.9 ± 0.4a	9.4 ± 0.1a	W14, W15
No. 02395–No. 96016	24.8 ± 0.7d	17.2 ± 0.3c	7.2 ± 0.4a	12.7 ± 0.5b	W14, W15
No. 02395–No. 4531	23.7 ± 1.6c	15.4 ± 0.6b	7.4 ± 1.8a	8.5 ± 0.6a	W14, W15
SWU63–No. 73290	20.7 ± 0.1b	21.1 ± 0.7b	8.5 ± 0.5a	7.0 ± 1.0a	W14
SWU63–No. 93237	21.5 ± 0.8c	16.6 ± 0.9b	11.4 ± 1.4a	9.5 ± 0.6a	W14
SWU63–Campina	21.3 ± 0.1c	12.3 ± 0.5ab	11.5 ± 0.6a	11.3 ± 1.5a	W14
SWU63–No. 96016	21.1 ± 1.5c	20.6 ± 0.4c	7.5 ± 0.5a	9.5 ± 1.0ab	W14
SWU63–No. 4531	20.4 ± 0.5cd	18.5 ± 2.1c	7.3 ± 2.6a	9.2 ± 1.4ab	W14
WH85–No. 73290	25.3 ± 3.0c	28.4 ± 2.0d	11.1 ± 1.2ab	9.5 ± 1.1a	W14
WH85–No. 93237	24.1 ± 2.6c	24.1 ± 1.1c	11.7 ± 0.6a	18.7 ± 1.2b	W14
WH85–Campina	25.4 ± 1.0c	18.5 ± 1.6b	11.9 ± 0.9a	12.1 ± 0.4a	W14
WH85–No. 96016	25.0 ± 1.2c	15.2 ± 0.5b	7.8 ± 2.0a	7.6 ± 0.6a	W14
WH85–No. 4531	22.1 ± 3.6b	22.5 ± 0.4b	8.1 ± 1.4a	7.7 ± 1.4a	W14
Zhongshuang11–No. 73290	21.2 ± 0.6b	20.7 ± 1.6b	10.5 ± 0.8a	10.4 ± 0.4a	W14
Zhongshuang11–No. 93237	20.1 ± 0.5d	17.7 ± 1.4c	12.4 ± 1.4a	14.6 ± 0.6b	W14
Zhongshuang11–Campina	21.5 ± 0.2c	10.9 ± 0.9b	8.1 ± 0.1a	7.8 ± 0.8a	W14
Zhongshuang11–No. 96016	21.2 ± 1.7d	14.5 ± 3.3c	7.5 ± 2.1a	9.4 ± 1.1ab	W14
Zhongshuang11–No. 4531	21.0 ± 0.6d	19.0 ± 0.8c	7.9 ± 1.8a	9.5 ± 1.3ab	W14

*“H” and “L,” respectively, denote the two types of high- and low-SNPP lines. The same lowercase letter in the same row indicates no significant difference at 0.05 probability level.*

For the four high-SNPP lines, the calculated maternal and xenia effects for the H×L crosses ranged from 0.41 to 1.00 and from 0 to 0.59, respectively, and the mean (0.70) of the former was significantly (*P* = 0.0019) larger than that (0.30) of the latter (**Figure 4**). It should be noted that, for the high-SNPP line No. 02395, the maternal and xenia effects were comparable for all of the five crosses in both years. Whereas for the other three high-SNPP lines, the maternal and xenia effects showed large variations in the different crosses. For the five low-SNPP lines, the calculated maternal and xenia effects of SNPP for the L×H crosses ranged from 0.40 to 1.00 and from 0 to 0.6, respectively, and the mean (0.88) of the former is much significantly (*P* = 1.8*E*-08) larger than that (0.12) of the latter. It should be noted that, the average maternal/xenia effect (0.88/0.12) of the five low-SNPP lines was a little significantly (*P* = 0.011) larger/smaller than that (0.70/0.30) of the four high-SNPP lines.

### Maternal Genotype, Embryo, and Cytoplasm Effects on SNPP

**FIGURE 5:**
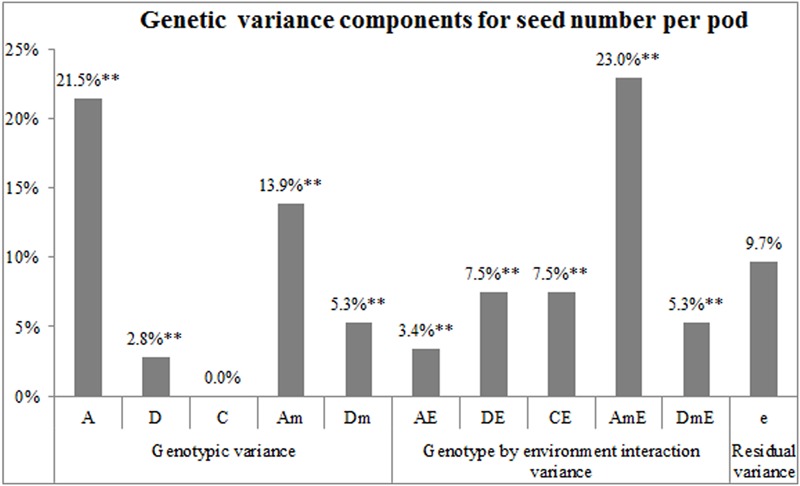
Estimation of the genetic variance components for SNPP using diploid seed embryo–cytoplasm–maternal model. The horizontal and vertical axes show the variance components and proportion, respectively. A, embryo additive; D, embryo dominance; C, cytoplasmic; Am, maternal additive; Dm, maternal dominance; AE, embryo additive interaction; DE, embryo dominance interaction; CE, cytoplasmic interaction; AmE, maternal additive interaction; DmE, maternal dominance interaction; and e, residual variance. ^∗∗^ represents the significant level of *P* = 0.01.

The diploid seed embryo–cytoplasm–maternal (GoCGm) model was also employed to quantitatively estimate the individual components of genetic effects (**Figure 5**). The results showed that numbers of seeds per pod for the nine lines were controlled by both the genetic main effect (*V*_A_ + *V*_D_ + *V*_C_ + *V*_Am_ + *V*_Dm_ = 43.5%) and genotype by environment interaction effect (*V*_AE_ + *V*_DE_ + *V*_CE_ + *V*_AmE_ + *V*_DmE_ = 46.7%). Among the effects of three sets of genetic systems (embryo, cytoplasm, and maternal plant), the maternal genotype (*V*_Am_ + *V*_Dm_ + *V*_AmE_ + *V*_DmE_ = 47.6%) accounted for the largest proportion of variance, followed by embryo effect (*V*_A_ + *V*_D_ + *V*_AE_ + *V*_DE_ = 35.2%), whereas cytoplasm effect (*V*_C_ + *V*_CE_ = 7.5%) was relatively small. Overall, these results indicated that the SNPP differences between the nine lines were mainly controlled by the maternal genotype and embryo effects, followed by a small cytoplasm effect.

### General and Special Combining Ability for SNPP

To investigate the combining ability for SNPP, the numbers of seeds per pod for the reciprocal hybrid F_1_ plants were also investigated. The results showed that the numbers of seeds per pod of the reciprocal hybrid F_1_ plants showed significant difference only for 7 of all the 20 reciprocal crosses (**Table 2**). In addition, these significant differences were usually small, which ranged from 1.1 to 4.6 with a mean of 2.8. These results suggested that the two types of lines had no or small cytological effect on SNPP. Therefore, the average number of seeds per pod for two reciprocal hybrid F**_1_** combinations was subjected to the analysis of combining ability for SNPP.

**Table 2:** The number of seeds per pod for the reciprocal hybrid F_1_ combinations.

Line combinations (H–L)	H×L	L×H	*P_t_*_-test_	Experiment code
No. 02395–No. 96016	23.5 ± 0.7	23.8 ± 0.3	0.492	W15
No. 02395–Campina	22.3 ± 1.5	22.5 ± 1.2	0.867	W15
No. 02395–No. 93237	22.4 ± 1.7	22.6 ± 1.0	0.803	W15
No. 02395–No. 4531	21.6 ± 0.9	19.0 ± 1.8	0.006	W15
No. 02395–No. 73290	22.5 ± 1.5	19.9 ± 1.4	0.002	W15
SWU63–No. 96016	17.0 ± 1.4	17.9 ± 2.2	0.531	W15
SWU63–Campina	15.1 ± 0.8	18.0 ± 1.4	6.5E-05	W15
SWU63–No. 93237	17.6 ± 0.9	18.7 ± 1.0	0.048	W15
SWU63–No. 4531	20.9 ± 1.6	21.5 ± 0.9	0.585	W15
SWU63–No. 73290	20.4 ± 1.5	20.1 ± 1.5	0.669	W15
WH85–No. 96016	15.9 ± 0.7	16.9 ± 1.6	0.203	W15
WH85–Campina	18.8 ± 1.7	19.9 ± 1.2	0.119	W15
WH85–No. 93237	15.4 ± 1.3	16.3 ± 1.4	0.130	W15
WH85–No. 4531	13.9 ± 0.6	16.3 ± 1.4	0.006	W15
WH85–No. 73290	15.4 ± 1.6	12.1 ± 1.2	1.9E-04	W15
Zhongshuang11–No. 96016	20.0 ± 1.7	19.3 ± 1.4	0.293	W15
Zhongshuang11–Campina	17.4 ± 0.6	18.0 ± 0.7	0.205	W15
Zhongshuang11–No. 93237	20.4 ± 1.2	20.4 ± 1.1	0.901	W15
Zhongshuang11–No. 4531	14.2 ± 0.2	18.8 ± 0.8	5.8E-04	W15
Zhongshuang11–No. 73290	18.3 ± 2.9	18.7 ± 2.2	0.709	W15

*“H” and “L,” respectively, denote the two types of high- and low-SNPP lines.*

The GCA effects of SNPP for the nine investigated lines ranged from -2.75 (WH85) to 3.17 (No. 02395) (**Table 3**), four and five of which were positive and negative, respectively. Except for the other five lines, the GCA effects of SNPP for No. 02395, No. 4531, No. 73290, and WH85 were statistically significant. It should be noted that the hybrid F_1_ combination between the parental lines both with high GCA effects of SNPP generally had high number of seeds per pod. As expected, the correlation between SNPP value of hybrid F_1_ and the total GCA effect of parents was highly significant (*r* = 0.82; *P* < 0.0001).

**Table 3:** The SCA between the four high-SNPP and five low-SNPP lines as well as the GCA for the nine lines.

	No. 96016	Campina	No. 93237	No. 4531	No. 73290	GCA
No. 02395	1.20**	0.23	0.11	-1.14*	-0.39	3.17***
SWU63	-1.72**	-2.33**	-0.95*	3.05***	1.95**	-0.12
WH85	-0.13	3.10***	-0.62*	-0.42*	-1.92**	-2.75***
Zhongshuang11	0.66*	-1.01*	1.47**	-1.48**	0.37	-0.29
GCA	0.44	0.16	0.38	-0.57*	-0.42*	

*^∗, ∗∗^, and ^∗∗∗^ indicate the significance level of *P* = 0.05, 0.01, and 0.001, respectively.*

The SCA effects of SNPP for the 20 combinations ranged from -2.33 to 3.10, 9 and 11 of which were positive and negative, respectively (**Table 3**). Except for the six combinations of No. 02395 and Campina, No. 02395 and No. 73290, No. 02395 and No. 93237, WH85 and No. 93237, WH85 and No. 96016, and Zhongshuang11 and No. 73290, the SCA effects of SNPP for the other 14 combinations were statistically significant. It should be noted that the SCA effects of SNPP for the Campina and WH85 combination was the largest (3.10), and the Campina×SWU63 combination was the smallest (-2.33). It should be noted that the hybrid F_1_ combination with high SCA effects of SNPP did not always have the high number of seeds per pod. As expected, the correlation between the SNPP value of hybrid F_1_ and the SCA effect of parental combination was moderate although significant (*r* = 0.57; *P* = 0.0036).

The analysis of variance showed that both the high- and low-SNPP parents and their interaction had significant effects on the numbers of seeds per pod for the reciprocal hybrid F_1_ combinations (Supplementary Table S4). The relative importance of GCA and SCA on SNPP were 63.0 and 37.0%, respectively, in accordance with their high and moderate correlation with SNPP value of hybrid F_1_ combinations. These results indicated that the number of seeds per pod for hybrid F_1_ was mainly determined by the GCA of parents, followed by the SCA of parental combination. It should be noted that the expected mean square of high-SNPP parent was much higher than that of the low-SNPP parent, which suggested that GCA of the parents were mainly attributed to the high-SNPP lines.

### Investigation on the Number of Ovules per Ovary

**FIGURE 6:**
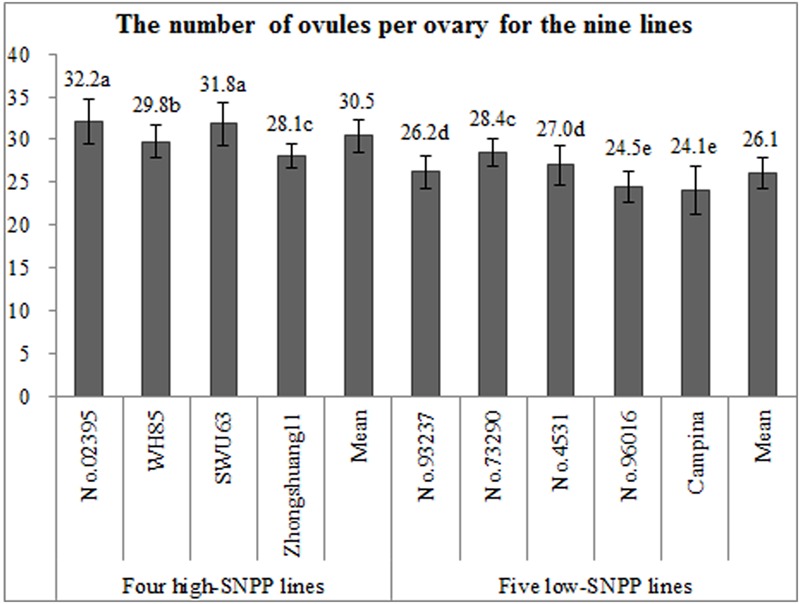
Comparison of the numbers of ovules per ovary among the nine rapeseed lines. The horizontal axis shows the four high-SNPP and five low-SNPP lines. The vertical axis shows the number of ovules per ovary. The same letter on the bar indicates no significant difference at the *P* = 0.05 probability.

The number of ovules per ovary was firstly investigated, because it is the predetermined condition for the formation of number of seeds per pod in rapeseed. Except for Zhongshuang11 and No. 73290, the numbers of ovules per ovary (27.9–32.2) for the four high-SNPP lines were significantly higher than those (24.1–28.3) for the five low-SNPP lines (**Figure 6**), and the mean (30.4) of the former was also significantly (*P* = 0.01) higher than that (26.0) of the latter. The difference between the average numbers of ovules per ovary for the four high-SNPP and five low-SNPP lines was 4.4, which explained 30.7% of the SNPP difference (14.3) between the two types of lines. As expected, the numbers of ovules per ovary of the nine lines were also highly significantly (*r* = 0.88; *P* = 0.0018) correlated with the corresponding numbers of seeds per pod (Supplementary Table S5).

It should be noted that the numbers of ovules per ovary were significantly larger than the corresponding numbers of seeds per pod for all of the nine lines (**Figures 1B, 6**), in accordance with the previous studies (Li et al., 2014; Li, 2015). This suggested that the number of ovules/seeds per ovary/pod should decrease from flowering to maturity.

### Continuous Investigation on the Number of Ovules/Seeds per Ovary/Pod

**FIGURE 7:**
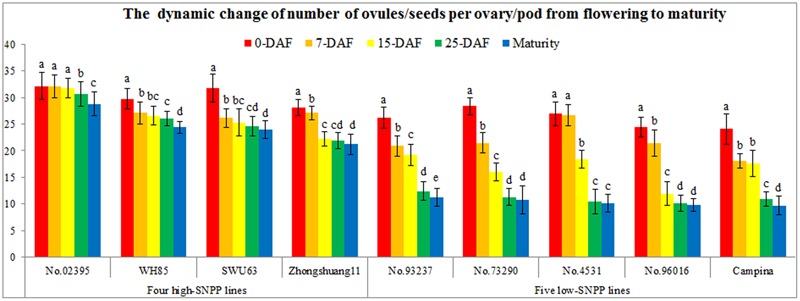
Continuous observation of the number of ovules/seeds per ovary/pod from flowering to maturity. The horizontal axis shows the different lines. The vertical axis shows the number of ovules/seeds. The legends of different colors discriminated the different stages. The same letter on the bar indicates no significant difference at the *P* = 0.05 probability.

To determine the decrease stages, the number of ovules/seeds per ovary/pod from flowering to maturity was serially investigated at 7-, 15-, and 25-DAF. From flowering to 25-DAF, the number of ovules/seeds per ovary/pod for all the nine lines showed an obvious decrease trend (**Figure 7**). Obviously, the decreased numbers (13.2–17.2) of the five low-SNPP lines were all larger than those (1.5–7.1) of the four high-SNPP lines, and the mean (15.0) of the former was also significantly (*P* = 8.2E-4) larger than that (4.7) of the latter. The difference between the means of decreased numbers for the four high-SNPP and five low-SNPP lines was 10.4, which accounted for 72.5% of the SNPP difference (14.3) between the two types of lines. Whereas from 25-DAF to maturity, the number of seeds per pod was basically stable, and only four lines (Campina, No. 02395, No. 93237, and WH85) showed a significant/small decrease from 1.2 to 1.9 (mean = 1.5). It should be noted that the decreased stage and speed were different for the different lines (**Figure 7**). From 0-DAF to 7-DAF, except for No. 02395 and No. 4531, the number of ovules per ovary for the other seven lines showed a significant decrease from 1.0 (Zhongshuang11) to 6.9 (No.73290), with a mean of 4.4. From 7-DAF to 15-DAF, the number of ovules/seeds per ovary/pod for the five lines (No. 4531, No. 73290, No. 93237, No. 96016, and Zhongshuang11) also showed a significant decrease from 1.7 (No. 93237) to 9.5 (No. 96016), with a mean of 6.0. From 15-DAF to 25-DAF, except for SWU63, WH85, and Zhongshuang11, the number of seeds per pod for the other six lines also showed a significant decrease from 1.1 (No. 02395) to 8.0 (No. 4531), with a mean of 4.8.

The observed decrease in the number of ovules/seeds per ovary/pod should be attributable to the failure of the fertilization of ovule (ovule failed to be fertilized) and the failure of the development of fertilized ovule (fertilized ovule failed to develop into seed). We speculated that the decrease of the number of ovules/seeds per ovary/pod from 0-DAF to 7-DAF, from 15-DAF to maturity, and from 7-DAF to 15-DAF should be due to failure of the fertilization of ovule, the failure of the development of fertilized ovule, and both, respectively.

### Investigation on the Fertilization of Ovules and the Development of Fertilized Ovules

**FIGURE 8:**
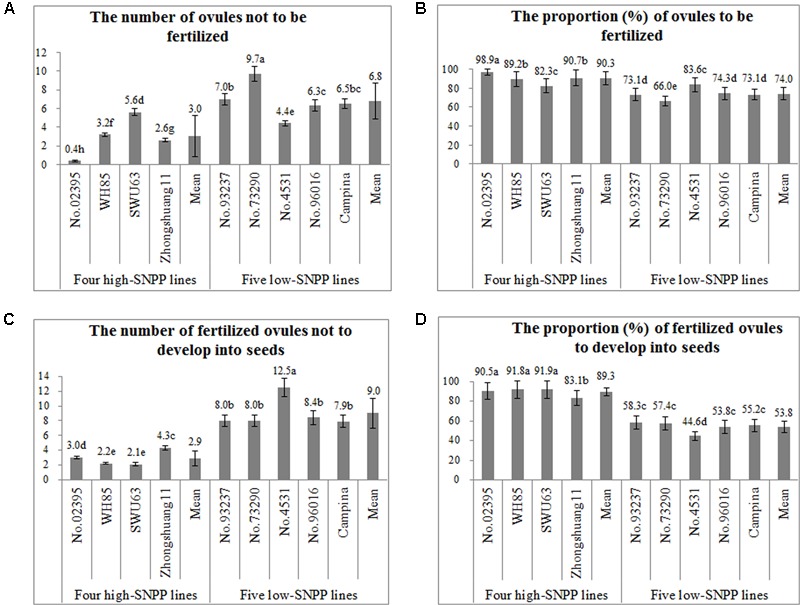
Comparison of the number and proportion related to the fertilization of ovule and the development of fertilized ovules among the nine rapeseed lines. The horizontal axis shows the four high-SNPP and five low-SNPP lines. The vertical axis shows the number **(A,C)** or proportion **(B,D)**. The same letter on the bar indicated no significant difference at the *P* = 0.05 probability.

To accurately discriminate the failure of the fertilization of ovule and the failure of the development of fertilized ovule, the number of fertilized/unfertilized ovules was observed and the corresponding proportion was then calculated for the nine lines (**Figures 8A,B**). The results showed that, except for SWU63 and No. 4531, the numbers of the unfertilized ovules (0.4–5.6) per ovary for the four high-SNPP lines were less than those (4.4–9.7) for the five low-SNPP lines, and the mean (3.0) of the former was also significantly (*P* = 0.031) smaller than that (6.8) of the latter. The difference between the average numbers of unfertilized ovules for the four high-SNPP and five low-SNPP lines was 3.8, which explained 26.8% of the SNPP difference (14.3) between the two types of lines. Accordingly, except for SWU63 and No. 4531, the proportions (82.3–98.9%) of ovules to be fertilized for the four high-SNPP lines were higher than those (66.0–83.6%) for the five low-SNPP lines, and the mean (90.3%) of the former was also significantly (*P* = 0.0095) higher than that (74.0%) of the latter. As expected, the proportions of ovules to be fertilized were highly significantly (*r* = 0.84; *P* = 0.0047) correlated with the numbers of seeds per pod for these lines (Supplementary Table S5).

Then, the number of fertilized ovules not to develop into seeds and the proportion of fertilized ovules to develop into seeds was calculated for the nine lines (**Figures 8C,D**). The results showed that the numbers of fertilized ovules not to develop into seeds (2.1–4.3) for the four high-SNPP lines were all much lower than those (7.9–12.5) of the five low-SNPP lines, and the mean (2.9) of the former was significantly (*P* = 9.5E-04) smaller than that (9.0) of the latter. The difference between the average numbers of fertilized ovules not to develop into seeds for the four high-SNPP and five low-SNPP lines was 6.1, which explained 42.4% of the SNPP difference (14.3) between the two types of lines. Accordingly, the proportions (83.1–91.9%) of fertilized ovules to develop into seeds for the four high-SNPP lines were all much higher than those (44.6–58.3%) for the five low-SNPP lines, and the mean (89.3%) of the former was also significantly (*P* = 1.1E-05) higher than that (53.8%) of the latter. As expected, the proportions of fertilized ovules to develop into seeds were also highly significantly (*r* = 0.96; *P* < 0.0001) correlated with the numbers of seeds per pod for these lines (Supplementary Table S5). The proportion of fertilized ovules to develop into seeds is affected by both internal (the nutritional and physiological status of the mother plant) and external (environmental conditions) factors as well as their interaction, which were rather complex and unable to be observed by cytological methods, and therefore had not been further investigated.

### Investigation on the Main Factors Related to the Fertilization of Ovules

**FIGURE 9:**
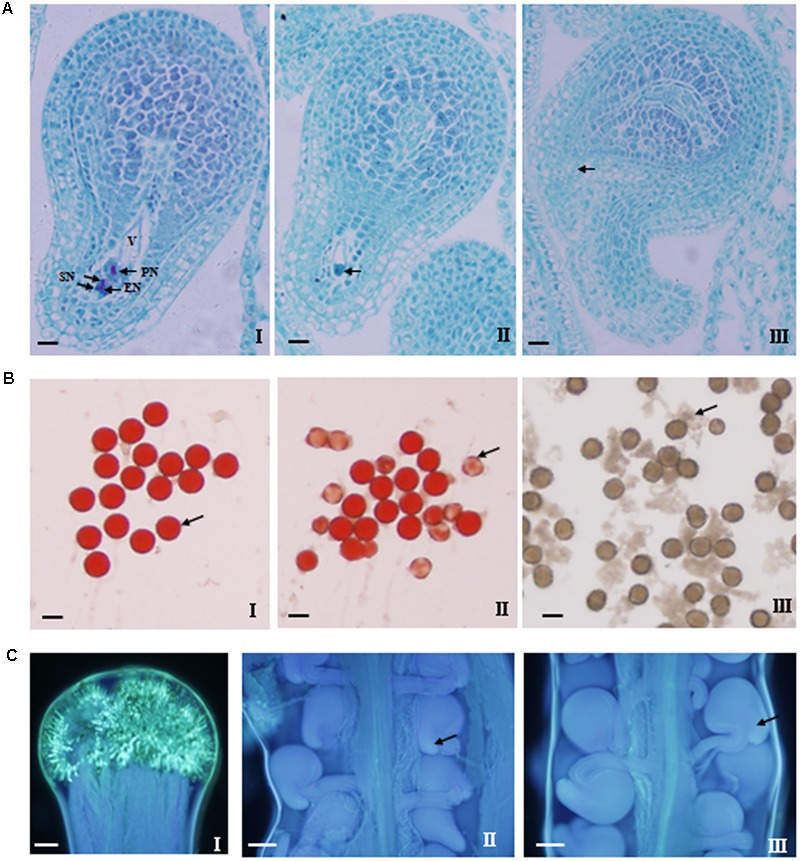
Cytological observations of ovule and pollen fertility as well as pollen germination and pollen tube growth and accession into ovary. **(A)** Normal embryo sac (I) and two types of abnormal embryo sac (II and III). A normal embryo sac consists of seven cells including eight nuclei: a single egg nucleus (EN), two polar nuclei (PN), two synergid nuclei (SN), and a central vacuole (V). The antipodal cells are degenerated or not clearly observed. The abnormal embryo sac exhibited two types of incomplete cellularization: (II) only two large cells were present in the embryo sac; (III) the embryo sac had not differentiated and lacked any visible cells. Bar = 10 μm. **(B)** Observation of pollen fertility (I and II) and pollen germination *in vitro* (III). The pollen grains with relatively deep dyeing and regular roundness were viable and fertile (arrow in I), while those with shallow dyeing and irregular shape were inviable and sterile (arrow in II). Bar = 10 μm. **(C)** Observation of pollen germination (I) on stigma and pollen tube accession into the micropyle (II–III). Pollen grains germinated on stigma at 72 h after hand-pollination (I), pollen tubes have passed into the micropyle, which also appear to be normal (arrow in II) and abnormal (arrow in III). Bar = 100 μm.

Because the proportion of ovules to be fertilized was mainly affected by ovule fertility, pollen fertility, and fertilization, etc., therefore, the related indexes were investigated (**Figure 9** and **Table 4**).

**Table 4:** The main indexes related to the proportion of ovules to be fertilized for the nine lines.

Type	Lines	Number of sterile ovules per ovary	Proportion of fertile ovules (%)	Pollen viability (%)	Pollen germination rate *in vitro* (%)	Pollen germination rate *in vivo* (%)	Pollen tube accession rate (%)
High-SNPP	No. 02395	1.4 ± 0.2f	95.5 ± 5.9a	94.2 ± 2.8abc	94.9 ± 5.6ab	100	100a
	WH85	3.0 ± 0.4e	90.0 ± 7.4b	95.5 ± 2.0ab	98.9 ± 1.0a	100	100a
	SWU63	5.5 ± 0.6b	82.8 ± 9.1cd	93.2 ± 2.6abc	96.6 ± 1.1ab	100	100a
	Zhongshuang11	1.4 ± 0.2f	95.0 ± 6.1a	92.8 ± 3.7c	91.1 ± 3.5b	100	100a
	Mean	2.8 ± 1.9	90.8 ± 5.9	93.9 ± 1.2	95.4 ± 3.3	100	100
Low-SNPP	No. 93237	4.9 ± 0.6c	81.3 ± 9.9d	95.0 ± 1.8ab	87.6 ± 0.9c	100	82.3 ± 3.7b
	No. 73290	6.8 ± 1.1a	76.2 ± 7.3e	96.3 ± 2.7a	67.7 ± 2.2d	100	85.1 ± 5.4b
	No. 4531	3.9 ± 0.4d	85.6 ± 6.4bc	93.5 ± 2.9abc	96.8 ± 3.8ab	100	100a
	No. 96016	5.7 ± 0.7b	76.8 ± 5.9e	88.3 ± 5.4d	96.8 ± 1.6ab	100	100a
	Campina	5.9 ± 0.9b	75.5 ± 3.9e	91.7 ± 4.1c	98.5 ± 1.5a	100	100a
	Mean	5.9 ± 1.1	79.1 ± 4.3	93.0 ± 3.1	89.5 ± 12.9	100	93.5 ± 9.0

*The different letters in the same column indicated no significant difference at the *P* = 0.05 probability.*

Firstly, the ovule fertility of the nine lines was investigated via the observation of the embryo sac morphology and structure by serial section. Both the normal and abnormal embryo sac structure were observed for all of the nine lines (**Figure 9A**). Generally, the normal embryo sac had seven cells including eight nuclei. However, the abnormal embryo sac showed the following two scenarios: only two large cells were present in the embryo sac or the embryo sac had not differentiated and had no any visible cells. Except for SWU63 and No. 4531, the numbers (1.4–5.5) of sterile ovules per ovary for the four high-SNPP lines were less than those (3.9–6.8) for the five low-SNPP lines, and the mean (2.8) of the former was also smaller than that (5.4) of the latter (**Table 4**). The difference between the average numbers of sterile ovules per ovary for the four high-SNPP and five low-SNPP lines was 2.6, which accounted for 18.2% of the SNPP difference (14.3) between the two types of lines. Correspondingly, except for SWU6 and No. 4531, the proportions (82.8–95.5%) of fertile ovules for the four high-SNPP lines were larger than those (75.5–85.6%) for the five low-SNPP lines, and the mean (90.8%) of the former was also significantly (*P* = 0.019) larger than that (79.1%) of the latter. Accordingly, from 0-DAF to 7-DAF, the decreased number of ovules per ovary for SWU63 was largest among the four high-SNPP lines, whereas the decreased number of ovules per ovary for No. 4531 was smallest among the five low-SNPP lines (**Figure 7**). As expected, the proportions of fertile ovules were highly correlated (*r* = 0.80; *P* = 0.01) with the numbers of seeds per pod for these lines (Supplementary Table S5).

Secondly, the pollen fertility of the nine lines was investigated by two evaluation index: pollen viability and germination rate *in vitro* (**Figure 9B**). The results showed that the pollen viability of the nine lines was all high (ranging from 88.3 to 96.3%), and the means (93.9 and 93.0%) for the four high-SNPP and five low-SNPP lines had no significant difference (**Table 4**). Except for No. 73290 (67.7%), the pollen germination rates *in vitro* of the other eight lines were also high (ranging from 87.6 to 98.9%) and the means (95.4% and 89.5%) for the four high-SNPP and five low-SNPP lines also had no significant difference. The results of both pollen viability and germination rate *in vitro* indicated that the pollen of all the nine lines should be fertile.

Thirdly, the pollen germination rate *in vivo* and pollen tube growth and accession were also investigated (**Figure 9C**). Although the pollen germination rates on stigma for the nine lines were all 100%, but the pollen tube accession rates of two low-SNPP lines (No. 73290 and No. 93237) were significantly lower than those for the other five lines (**Table 4**). In fact, the calculated proportions of fertile ovules being successfully fertilized for the two lines were also significantly lower than those for the other seven lines, suggesting the fertilization obstacles in the two lines (**Figure 7**).

## Discussion

The number of seeds per pod for the nine lines exhibited a wide range (**Figure 1B**), which almost represented its large variation in rapeseed (Zhang et al., 2008; Chen, 2014). The average heterozygosity of the nine lines was nearly 0 (**Figure 2A**), which suggested that they should be highly pure. In addition, both the gene diversity and PIC for the nine lines were relatively high and comparable to those estimated by tens to hundreds of lines in the previous studies (Lai et al., 2013; Zhou et al., 2015), which suggested that they had a rich genetic diversity. More importantly, the nine lines also showed large genetic distances (**Figure 2B**) and very low kinship coefficients (**Figure 2C**), which suggested that they had large genetic variation. Therefore, the nine lines should be ideal representative materials that could be used for the further research on SNPP.

The major seed traits in rapeseed include seed number, seed weight, and seed quality (i.e., contents of glucosinolate, protein, oil, and fatty acid compositions, etc.). Of these, the glucosinolate and erucic acid contents in the seeds are well known to be controlled by the maternal and embryo genotype, respectively (Liu, 2000); while seed weight and oil content have been proved to be mainly controlled by maternal genotype in our previous studies (Wang et al., 2010; Li N. et al., 2015). However, the relative importance of maternal genotype, embryo, and cytoplasm effects as well as maternal and xenia effects on SNPP have not been investigated and thus unclear in rapeseed. To our knowledge, the current study is the first report on the accurate estimation of the relative contributions of these genetic effects on SNPP in rapeseed.

The results of self- or cross-pollination between the four high-SNPP and five low-SNPP lines (**Figure 3**) showed that the natural variation of SNPP was mainly controlled by maternal effect, followed by xenia effect. Although many studies have investigated the xenia effects in plants, almost all of these were on seed/fruit size/weight and quality traits (Jiang et al., 2007; Zhu et al., 2009; Wang et al., 2010; Zhou et al., 2011; Li N. et al., 2015), and only a few involved seed number. Unfortunately, the relative contribution of xenia vs. maternal effects on seed number has not been estimated in these studies and thus is not able to compare with the current study (Wallace and Lee, 1999; Jiang, 2004). It should be noted that both the maternal and xenia effects for 12 crosses have comparable contributions on SNPP. This might be caused by the disharmony between the pollen grain/pollen tube including the sperm cells with the various sporophytic maternal tissues and the cells of the female gametophyte (Dresselhaus and Franklin-Tong, 2013; Bleckmann et al., 2014; Zhang et al., 2017). In addition, analysis of the data using the diploid seed embryo–cytoplasmic–maternal (2nGoCGm) model (Zhu, 1997) further showed that the natural variation of SNPP was mainly governed by maternal genotype and embryo effects, followed by a small cytoplasm effect. In fact, many studies have estimated the relative contribution of embryo, endosperm, cytoplasm, and maternal genotype effects on a large range of seed traits, especially in crop plants (Li et al., 2000; Shi et al., 2000; Ning et al., 2006; Wang et al., 2007). However, none of these studies has involved seed number and thus is not able to compare with the current research. Maternal genotype and cytoplasm effects can be attributed to maternal effect, while embryo effect can be attributable to both maternal and xenia effects. The estimated proportions of maternal genotype, embryo, and cytoplasm effects were also highly accordant with those for maternal and xenia effects. The GCA and SCA effects estimated in the current study ranged from nearly 0 to about (±) three seeds per pod, which were comparable to the previous studies (Sabaghnia et al., 2010; Tian et al., 2015; Muhammad et al., 2017). The analysis of variance showed that the SNPP of hybrid F_1_ was mainly determined by GCA of parents, followed by SCA of parental combination. This suggested that SNPP is mainly governed by additive effects, followed by non-additive effects, a conclusion in agreement with the previous studies (Zhang et al., 2008; Sabaghnia et al., 2010; Xing et al., 2014). Especially, the GCA for No. 02395 was much larger than the other eight lines, which should be used as a good combiner for SNPP.

The main biological processes that may affect the number of seeds per pod are basically known in rapeseed (Li et al., 2014), however, which processes are responsible for the natural variation of SNPP as well as their relative contributions are poorly known. To our knowledge, this is the first systematic study to accurately/quantitatively estimate the relative contributions of these biological processes to the natural variation of SNPP in rapeseed.

The systematic analyses and comparisons showed that the SNPP difference between the high- and low-SNPP lines was due to the accumulative differences in its components: the number of ovules, the proportion of fertile ovules, the proportion of fertile ovules to be fertilized, and the proportion of fertilized ovules to develop into seeds (**Figures 6–9** and **Table 4**). Of these, the number and fertility of ovule are determined by the ovule differentiation and development processes before flowering (Yang et al., 2016), which both are fully controlled by maternal genotype. The fertilization rate of fertile ovule is mainly determined by the pollen fertility as well as the interaction between pollen grain/pollen tube including the sperm cells with the various sporophytic maternal tissues and the cells of the female gametophyte (Dresselhaus and Franklin-Tong, 2013), which are controlled by both maternal and xenia effect. The survival rate of fertilized ovules is determined by the seed development process after fertilization (Li et al., 2014), which is controlled by both the maternal and embryo genotype. Therefore, the results of both genetic and cytological analyses are highly accordant. These results provide solid evidences and systematic insights to further understand the mechanisms underlying the natural variation of SNPP, which will facilitate the development of high-yield cultivars in rapeseed.

It should be noted that the number of seeds per pod for the elite cultivar Zhongshuang11 is much more less than the number of ovules per ovary as well as its maximum reported in the current (**Figure 6**) and previous (Li et al., 2014; Li, 2015) studies. In addition, both the fertilization rate of ovules and the survival rate of fertilized ovules for the elite cultivar Zhongshuang11 were less than the maximum observed in the current studies (**Figure 8**). These results suggested the great potential of the genetic improvement of SNPP in rapeseed. No significant negative correlation was observed among these components (i.e., number of ovules per ovary, proportion of fertile ovules, proportion of ovules being successfully fertilized and proportion of fertilized ovules successfully developing into seeds) of SNPP (Supplementary Table S5), which suggested that the SNPP could be improved by the simultaneous increase of several components. More importantly, several lines with favorable characters related to SNPP have been found, such as high number of ovules (No. 02395 and SWU63), good fertility of ovules (No. 02395 and Zhongshuang11), high fertilization rate of ovules (No. 02395), and high survival rate of fertilized ovules (WH85 and SWU63). These lines could be used as the donor parents in the genetic improvement of SNPP. Furthermore, the lines with contrasting characters related to SNPP could be used to construct the genetic populations to map and further clone the underlying genes that are acting at the specific stages. In fact, a major QTL for SNPP (*qSN.A6*) had been mapped using the BnaZNF_2_ population derived from the two (Zhongshuang11 and No. 73290) of the nine lines (Shi et al., 2015). The following study showed that *qSN.A6* was controlled by maternal genotype effect due to the ovule sterility (Yang et al., 2016). As expected, its additive effect (5.3 seeds per pod) was approximately equal to the difference (5.4 ovules per ovary) between the numbers of sterile ovules per ovary for the two parents (**Table 4**). The high consistency between the results of genetic analysis and cytological observation of the parental lines with the mechanism and effect of the underling QTL highlights the usefulness of these lines for further study.

## Author Contributions

HW and JS designed the experiments. GL and XW provided the research materials. YY performed the genetic experiments. JZ collected the phenotypic data. YY and YW performed cytological experiments. YY and JS analyzed the data. JS, YY, and YW wrote the manuscript.

## Conflict of Interest Statement

The authors declare that the research was conducted in the absence of any commercial or financial relationships that could be construed as a potential conflict of interest.

## Footnotes

1 http://apps.fas.usda.gov/psdonline/

2 http://ibi.zju.edu.cn/software/qga/index.htm
